# Licochalcone D mitigates intracerebral hemorrhage-induced ferroptosis of neurons through COX2 inhibition

**DOI:** 10.3389/fphar.2025.1566724

**Published:** 2025-07-08

**Authors:** JiaLi Song, HuiYing Qiao, ShunLi Dong, JingMei Tang, Bai Li, Xi Zhou, Shan Lv, Rong Lv

**Affiliations:** ^1^ Department of Geriatrics, Suzhou Ninth People’s Hospital, Suzhou, Jiangsu, China; ^2^ Department of Pharmacology, College of Pharmaceutical Sciences, Soochow University, Suzhou, Jiangsu, China; ^3^ Department of Geriatrics, The First Affiliated Hospital of Nanjing Medical University, Nanjing, Jiangsu, China

**Keywords:** licochalcone D, COX2, intracerebral hemorrhage, secondary brain injury, ferroptosis

## Abstract

**Objective:**

This study aims to assess LCD’s neuroprotective pharmacological effects against SBI post-ICH and identify its ferroptosis-inhibiting targets.

**Methods:**

Animal models of ICH and cellular models of SBI were established. Subsequently, gradient concentrations of LCD were administered at both the animal and cellular/molecular levels. The extent of ICH injury was evaluated using a range of methods, including CCK8 assay, Flow Cytometry, quantification of CAT and MDA, CI staining, Western blot, and HE staining. The SWISS TARGET prediction tool and molecular docking were utilized to confirm LCD’s target pathway and its binding site on COX2. Quantification of ferroptosis-executing proteins, BODIPY ROS staining, quantification of PGE2, MDA, and CAT were observed to assess the pharmacological effects, trends in ferroptosis influence, and to elucidate the underlying pathway mechanism.

**Results:**

Pretreatment with LCD can improve the state of SBI before the induction of an ICH model. Compound target prediction analysis revealed 102 differentially expressed genes (*p* < 0.05) associated with the drug target of LCD, with COX2 exhibiting the most significant expression. Furthermore, we found that LCD intervention suppressed COX2 expression, and pretreatment with COX2 overexpression in the ICH model group negated the pharmacological effects, of LCD on neuronal cell ferroptosis and SBI. It is proposed that by targeting COX2 through early LCD administration in ICH, ferroptosis in nerve cells can be reduced and SBI outcomes can be improved. To further elucidate the mechanism of targeting COX2, we found that PGE2, a downstream metabolite of COX2, is also regulated by LCD. By screening its impacts on the EP receptor family (EP1, EP2, EP3, EP4), it was found that COX2 is specifically targeted and suppressed by LCD pretreatment prior to ICH modeling, which further inhibits the PGE2/EP1 pathway, thereby reducing ferroptosis-specific lipid peroxidation.

**Conclusion:**

LCD pretreatment reduces ferroptosis in neurons and alleviates SBI after ICH by blocking the COX2/PGE2/EP1 pathway. Early LCD use may improve SBI, highlighting its potential as a pharmacological option for ICH outcomes.

## Introduction

Intracerebral hemorrhage (ICH), constituting 10%–15% of global stroke incidence, exhibits a dismal prognosis, with up to 50% death within 30 days. ICH entails cerebrovascular breach triggering parenchymal compression via hydrostatic pressure propagation, culminating in primary brain insult (Li et al., 2018). After primary hemorrhagic brain injury, the hematoma initiates intracranial reactive processes like inflammation, oxidative stress-mediated cell death, mitochondrial dysfunction, and BBB permeability, culminating in secondary neurological damage (Hu et al., 2016; Chen et al., 2018). Early surgical hematoma removal is the main treatment, but its success and clinical benefits are limited (Mendelow et al., 2013). Over the past few decades, the bulk of experimental research has focused on understanding secondary injury mechanisms after ICH to identify new therapeutic targets.

After ICH, a cascade of events initiates secondary brain injury (SBI). Hemoglobin and its metabolites from ruptured red blood cells decompose into iron, carbon monoxide, and biliverdin. These breakdown products strongly activate microglia, which phagocytose debris and harmful substances while also triggering a robust inflammatory response by releasing pro-inflammatory cytokines (Dai et al., 2022). Additionally, iron released into the bloodstream is transported into cells and converted to Fe^2+^, which can induce lipid auto-oxidation by generating ROS through iron-catalyzed enzymes, promoting ferroptosis (Chen et al., 2020). At the molecular scale, the equilibrium of cysteine supply, the biosynthetic productivity of glutathione (GSH), and the functional integrity of glutathione peroxidase 4 (GPX4) constitute the core regulatory mechanisms for ferroptosis suppression (Seibt et al., 2019). Also, NADPH oxidase (NOX), a key enzyme in reactive oxygen species (ROS) production, is activated. The generated ROS trigger lipid peroxidation reactions, producing alkyl radicals and other reactive species (Xie et al., 2020). Accumulation of lipid peroxides and free radicals not only induces ferroptosis but also activates and polarizes macrophages, thereby sustaining and amplifying inflammatory factors production. These interconnected mechanisms synergistically drive the development and progression of SBI, ultimately causing lasting neurological damage. Recent studies highlight the therapeutic potential of ferroptosis inhibition in providing neuroprotection during acute and chronic ICH stages (Zhang et al., 2018). Therefore, targeting more potent ferroptosis inhibitors as a strategic approach may disrupt the harmful mechanisms underlying SBI.

Cyclooxygenase 2 (COX2) is an oxygenase that generates superoxide upon activation. Prostaglandin E2 (PGE2) is a primary metabolite of COX2 activity in the cerebral parenchyma (Kikuchi et al., 2019; Li et al., 2025). Neuronal damage and oxidative stress may result from prostaglandins (Plastira et al., 2020) and/or inflammation linked to free radicals (Blaker et al., 2019; Ruiz-Roso et al., 2018). PGE2 serves as a retrograde messenger in hippocampal synaptic pathways (Maingret et al., 2017), primarily via four Prostaglandin E Receptor (EP receptors) (Narumiya et al., 1999). Prostaglandin E Receptor 1 (EP1) promotes Ca^2+^ mobilization, Prostaglandin E Receptor 2 (EP2) and Prostaglandin E Receptor 4 (EP4) elevate cyclic adenosine monophosphate through GS proteins, and Prostaglandin E Receptor 3 (EP3) inhibits adenylyl cyclase via GI proteins. It has been discovered that (Cruz et al., 2012; Ma et al., 2017), in chronic neuropathic pain, COX2 and PGE2 influence EP1and EP4. In the Partial sciatic nerve ligation (PSNL) model-induced chronic neuralgia, the synthesis of Brain-derived neurotrophic factor (BDNF) within dorsal root ganglion (DRG) sensory neurons is dynamically regulated, driving the pathogenesis of neuropathic pain. Nevertheless, the exact role of the COX2/PGE2 pathway and its inhibitors in neuronal cell ferroptosis following ICH remains poorly understood.

Licochalcone D (LCD) belongs to the class of chalcone-type flavonoids, isolated from the traditional Chinese medicinal herb *Glycyrrhiza inflata Batalin [Fabaceae]*. A potent natural compound with proven anticancer, antioxidant, and anti-inflammatory action (Seo et al., 2019; Zhang et al., 2024a; Maharajan et al., 2021). Recent research has demonstrated that LCD may mitigate the neurodegenerative symptoms in Parkinson’s disease patients (Oh et al., 2023). However, the role of LCD in ICH remains unexplored.

In the *in vitro* part of our study, LCD exerts pharmacological effects, revealing its potential as a COX2 inhibitor and the molecular mechanism by which LCD prevents ICH-mediated ferroptosis, suppresses oxidative stress, and alleviates inflammatory responses. This contributes to the development of LCD-based drugs and their application in ICH treatment.

## Materials and methods

### *In Vitro* Model of Cellular ICH and treatments

Cell death induction by CoCl_2_ utilized as an *in vitro* model to simulate ischemia-hypoxia conditions in SBI following ICH, as previously reported (Toro-Urrego et al., 2019). The PC12 rat adrenal pheochromocytoma cell line and SH-SY5Y human neuroblastoma cells, obtained from the Shanghai Cell Resource Center of the Chinese Academy of Sciences, were cultured in Dulbecco’s Modified Eagle’s Medium (DMEM, Gibco) supplemented with 10% heat-inactivated fetal bovine serum (FBS, Gibco) and a 1% antibiotic cocktail containing penicillin and streptomycin. Growth conditions were maintained at 37°C in a 5% CO2-humidified atmosphere for cultural development. A total of 9,000 or 2 × 10^5^ PC12 or SH-SY5Y cells were seeded per well in Gletrex™-coated 96-well or 100 mm tissue culture microplates. After allowing them to grow for a period of 24-h, the culture medium was replaced with serum-free medium. For the injury group, the cells were exposed to 400 μg/mL of CoCl_2_ (7,791–13-1, MCE Co., China) to induce hypoxia for 24 h. Licochalcone D (LCD, purity: >99.8%) was purchased from MCE (HY-N4187, United States). Model group cells received preliminary treatment with different concentrations (for PC12 rat adrenal pheochromocytoma cells, a concentration of either 2 μM or 5 μM was selected; for SH-SY5Y neuroblastoma cells, a concentration of either 5 μM or 10 μM was chosen) of LCD 2 h before CoCl_2_ modeling. Ferrostatin-1 (FE-1; HY-100579, MCE, United States) at 1 μM was also used for pretreatment 2 h before CoCl_2_ modeling, as per the manufacturer’s instructions. COX2 was overexpressed in cells by transfecting them with the COX2 plasmid (COX2 NM_017232 in pcDNA3.1, sourced from Nanjing Juhua Xiong Biotechnology Co., Ltd., China) using Lipofectamine 3,000 (Invitrogen, United States). The medium was refreshed with basal medium after 24 h, followed by LCD and CoCl_2_ treatment.

### Cell viability assay

The PC12 rat adrenal pheochromocytoma cells or SH-SY5Y neuroblastoma cells were plated in 96-well dishes and incubated for 24 h. LCD solutions of varying concentrations were incorporated into the serum-deficient medium for a 24-h period. (For PC12 rat adrenal pheochromocytoma cells, a concentration of either 0–20 μM was selected; for SH-SY5Y neuroblastoma cells, a concentration of either 0 μM or 20 μM was chosen). Cell viability was measured using the Enhanced Cell CCK8 assay (C0043, Beyotime Biotechnology Co., China) according to the manufacturer’s instructions. The 96-well plate was placed on the microplate reader (Molecular Devices, San Jose, CA, United States), the absorbance of the samples was assessed at 450 nm. Each well’s optical density (OD) was meticulously documented.

### Flow cytometry

The PC12 rat adrenal pheochromocytoma cells or SH-SY5Y neuroblastoma cells (2 × 10^5^ cells) were allocated into these groups: 1) control group; 2) CoCl_2_-induced model group; 3) CoCl_2_ + LCD pretreatment group; 4) CoCl_2_ + FE-1 pretreatment group; 5) COX2-OE + CoCl_2_ + LCD group. (The drug concentrations and the plasmid transfection methods utilized were in accordance with those described in the preceding section titled “*In vitro* Model of Cellular ICH and Treatments”). As per the manufacturer’s instructions, the cell suspension was then incubated with DCFH-DA (EX = 488 nm, EM = 525 nm; S0033M, Beyotime Biotechnology) for 30 min. The cells were then resuspended for flow cytometry to measure intracellular reactive oxygen species (ROS). The fluorescence intensity of FITC in flow cytometry outcomes served as a metric for gauging intracellular ROS concentrations.

### Measurement of lipid ROS

Lipid ROS levels were assessed using established methods (Yoshida et al., 2003). Briefly, cells were plated on glass slides for further experiments. The drug concentrations and plasmid transfection methods for PC12/SH-SY5Y cells (2 × 10^5^ cells) adhered to the protocols outlined in the “*In vitro* Model of ICH and Treatments”. The experimental grouping was designed with reference to “Flow Cytometry”. Cells subjected to treatment were then incubated with 1 mM of the BODIPY 581/591 C11 dye (D3861, Invitrogen, Carlsbad, United States) for a duration of 30 min within an incubator. Fluorescence microscopy (Olympus, fv3000) was used to image lipid ROS levels in one group of cells, with the resulting fluorescence pictures analyzed by a researcher who was unaware of the experimental conditions. Oxidized and reduced dye fluorescence intensities were measured using FITC (EX/EM: 488/525) and BODIPY RED (EX/EM: 581/591), respectively. The FITC/BODIPY RED fluorescence intensities served as a readout for cellular lipid peroxidation. The fluorescence intensity measurements were quantified using Image J software. Fluorescence density quantifications were normalized prior to plotting. Prior to plotting, the fluorescence density (FITC/RED) for each group was quantified.

### Western blot

The drug concentrations and plasmid transfection methods for PC12/SH-SY5Y cells (2 × 10^5^ cells) adhered to the protocols outlined in the “*In vitro* Model of ICH and Treatments”. The experimental grouping was designed with reference to Flow Cytometry, while the *vivo* models and their respective groupings were based on the protocols described in the animal experimentation section. Cells and tissues samples were homogenized using a Precellys Evolution homogenizer (Bertin Technologies) in 500 μL of protein extraction buffer (50 mM HEPES, 100 mM NaF, 150 mM NaCl, 10 mM Na_4_P_2_O_7_, 0.5% sodium deoxycholate, 1% Triton X-100, 10 mM EDTA, 0.1% SDS, ddH_2_O), supplemented with 1:100 protease (Thermo Fisher, 1862209) and phosphatase (Thermo Fisher, 1862495) inhibitor cocktails. The lysate was filtered 5–8 times using a 25G needle and centrifuged twice (20,800 g, 10 min, 4°C). Protein concentration was measured with a Bradford assay (Coomassie Plus reagent, Thermo Fisher 23,236) in a 96-well plate, with absorbance at 596 nm recorded via Spectramax to create a standard curve. The protein samples were subsequently blended with a 4× NuPAGE loading buffer from Invitrogen and a dash of 5% β-mercaptoethanol, resulting in a concentration of 20 μg per microliter. The mixture was then zapped at 95°C for a full 5 minutes before getting spun at 20,800 g for a solid 2-min whirl. Protein samples (10–20 μg) were separated via 7.5%–12% SDS-PAGE gels and transferred to PVDF membranes (Bio-Rad Laboratories, Hercules, CA). The separation and transfer processes were carried out under standard denaturing conditions to ensure optimal protein migration and membrane binding. The membrane was then placed in a container and covered with a 3% BSA solution, allowing it to incubate at room temperature (RT) for 1 h. The membranes were incubated overnight at 4°C with primary antibodies diluted in 3% BSA, followed by three TBST washes (Tris-buffered saline with Tween 20), 8 min per wash. Next, the membranes underwent incubation with secondary antibodies at RT for 1 h, followed by another three washes with TBST (8 min each). Protein signals were detected using the enhanced chemiluminescence (ECL) method (Beyotime Biotechnology, Shanghai, China). The ChemiDoc system (Bio-Rad) visualized the bands, which were then quantified via ImageJ densitometry. β-actin served as an internal control. The following antibodies were used at specified dilutions: p53 (YP0205 from Immunoway at 1:500), BAX (50599-2-Ig from Proteintech at 1:2000), BCL2 (68103-1-Ig from Proteintech at 1:2000), COX2 (66351-1-Ig from Proteintech at 1:1000), GPX4 (67763-1-Ig from Proteintech at 1:1000), EP1 (YT5447 from Immunoway at 1:500), EP2 (YT1566 from Immunoway at 1:500), EP3 (YT1569 from Immunoway at 1:500), EP4 (YT1570 from Immunoway at 1:500), and β-actin (goat anti-β-actin from Santa Cruz Biotechnology at 1:5000).

### Quantitative real-time PCR

The drug concentrations and plasmid transfection methods for PC12/SH-SY5Y cells (2 × 10^5^ cells) adhered to the protocols outlined in the “*In Vitro* Model of ICH and Treatments”. The experimental grouping was designed with reference to Flow Cytometry, while the *vivo* models and their respective groupings were based on the protocols described in the animal experimentation section. With Trizol, 1 μg of total RNA was extracted from the cells and tissues, subsequent treatment with DNase I (P0346S, Beyotime, China) efficiently eliminated DNA contamination. RNA purity was assessed through the A260/A280 ratio measurement with a UV spectrophotometer from Thermo Fisher Scientific Inc., United States, which indicated an optimal range between 1.8 and 2.0. 500 ng of RNA was reverse-transcribed into cDNA using a 10 μL reaction mixture containing PrimeScript™ RT Master Mix (RR036A, Takara, Japan) under the following conditions: 15 min at 37°C, followed by 5 s at 80°C. Quantitative PCR was executed within a 10 μL reaction blend with the application of TB Green^®^ Premix Ex Taq™ II (RR820A, Takara, Japan), with primer concentrations set at 0.4 μM. Thermal cycling parameters: 95°C for 30 s, then 40 cycles of 95°C (5 s) and 60°C (30 s). We have incorporated the use of β-actin as an internal control and established a negative control to monitor and detect potential contamination. ΔΔCt method assessed gene expression levels. Quantification of *IL-6, IL-1β*, and *TNF-α* levels involved PCR. The following primers were used in this study: *β-actin* 5′-CAC​GAT​GGA​GGG​GCC​GGA​CTC​ATC-3′ (forward) and 5′-TAA​AGA​CCT​CTA​TGC​CAA​CAC​AGT-3′ (reverse); *IL-6* 5′-CCC​AAC​TTC​CAA​TGC​TCT​CCT​AAT​G-3′ (forward) and 5′-TTG​CCG​AGT​AGA​CCT​CAT​AGT​GAC-3′ (reverse); *IL-1β* 5′-CCC​AAA​CAA​TAC​CCA​AAG​AAG​AAG​ATG-3′ (forward) and 5′-CTG​CTT​GAG​AGG​TGC​TGA​TGT​AC-3′ (reverse); *TNF-α* 5′-GCG​TGT​TCA​TCC​GTT​CTC​TAC​C-3′ (forward) and 5′-AGA​GCC​ACA​ATT​CCC​TTT​CTA​AGT​TAG-3′ (reverse).

### Detection of MDA and CAT

The drug concentrations and plasmid transfection methods for PC12/SH-SY5Y cells (2 × 10^5^ cells) adhered to the protocols outlined in the “*In Vitro* Model of ICH and Treatments”. The experimental grouping was designed with reference to Flow Cytometry, while the *vivo* models and their respective groupings were based on the protocols described in the animal experimentation section. Tissue samples underwent pre-processing steps tailored for Western blot, with protein concentrations standardized through BCA quantification to facilitate accurate comparisons across samples. Adhering to standard protocols, positive, negative, and blank controls were meticulously established. The levels of Malondialdehyde (MDA) were standardized and reported in nanomoles per milligram of protein (nmol/mgprot), whereas the Catalase (CAT) enzyme activity was measured in units per milligram of protein (U/mgprot). Markers of oxidative stress damage, MDA and CAT were extracted and measured using respective kits from Nanjing Jiancheng Bioengineering Institute, China (MDA kit: A003-1; CAT kit: A007-1-1). The kits were utilized in compliance with the manufacturer’s guidelines.

### Animal experiments

8 weeks old Sprague-Dawley (SD) rats, around 250 g each, were obtained from Vitalriver Company (NanJing, China). Rats were housed in a sterile environment with a 12-h light and dark schedule, within a constant temperature range of 19–23°C and humidity levels of 55% ± 10%. And all the animal experiment procedures were approved by The Institutional Review Board of Nanjing Medical University (License code: IACUC-2209029). A total of 20 rats were enrolled in the study (n = 5). After a week of adjustment, participants were randomly divided into four groups, these included: a control group receiving PBS injections, an ICH model group, an ICH group treated with low-dose LCD (5 mg/kg), and an ICH group receiving high-dose LCD (20 mg/kg). (materials were uniformly collected 24 h after ICH induction, and LCD was administered via tail vein injection 2 h prior to ICH modeling). Ultimately, six rats that failed to exhibit significant hematoma were excluded from the study, as they did not display neurological deficits post-surgery. (Post-euthanasia examination revealed the absence of hematoma in their brains). This exclusion ensured a final sample size of three rats per group (n = 3).

The ICH model was created by injecting autologous blood into the right basal ganglia using stereotactic methods, following established protocols (Wang et al., 2018). Rats were anesthetized with 4% isoflurane and positioned on a stereotactic head frame (Kopf Instruments, Tujunga, CA, United States), and kept at 37°C with a heating pad. Artificial tear ointment maintained ocular moisture during the process. Blood (60 μL) was extracted from the tail artery into a non-heparinized tube, then swiftly transferred to a Hamilton syringe via a 27-gauge needle. A transverse cut was performed to reveal the cranium completely, allowing for a 1 mm opening to be bored directly into the bone, and the Hamilton syringe was placed into the right basal ganglia following stereotactic coordinates (0.2 mm posterior, 2.2 mm lateral). Subsequently, a total of 30 μL of autologous blood was infused at a rate of 2 μL/min using a microinfusion pump (Stoelting, Harvard Apparatus, Holliston, MA) (5 μL initially at 3.0 mm below the dura, followed by 25 μL at 3.5 mm below the dura after 5 min). The needle was retained for 10 min before withdrawal, sealed with bone wax, and rats were sutured. They recovered on a 37°C pad; control surgeries matched the procedure without blood.

Post a day’s interval, the rodents were anesthetized using a blend of isoflurane and oxygen (a 4% isoflurane concentration). In no time, approximately 30 mg of their 0.5 cm^3^ massive heads were swiftly lopped off. The scalp was slit open, revealing the exposed skull. Carefully, the skull was detached along the suture lines, thus unveiling the brain tissue, which was then promptly chilled on ice. Subsequently, the brain tissue surrounding the hematoma was speedily extracted for more in-depth studies. Throughout the sampling, the rat brain tissues were preserved at a low temperature whenever feasible.

### IHC and TUNEL analysis of tissue specimens

The *vivo* models and their respective groupings were based on the protocols described in the animal experimentation section. Brain tissues around the hematoma area were immediately fixed in 4% paraformaldehyde for 24 h, subsequently embedded in paraffin and sliced to 5 μm using a microtome. Following rehydration, endogenous peroxidase activity was blocked by treating the sections with 3% H_2_O_2_ for 10 min. Antigen retrieval was carried out in an autoclave for 3 min to expose epitopes. Sections were blocked with goat serum for 45 min, then incubated overnight at 4°C with a 1:200 COX2 primary antibody. After PBS rinses, a secondary antibody was applied and incubated at room temperature for 60 min, followed by washing and 10-min DAB staining in the dark. Nuclei were visualized by staining with hematoxylin for 5 min. Following dehydration via an alcohol gradient and clarification with dimethylbenzene, the slides were coverslipped using neutral balsam and analyzed with an automated scanning microscope (PreciPoint, Freising, Germany). To assess the level of neuronal apoptosis, we conducted TUNEL (green) staining with the Apoptosis Detection Kit from Roche, United States, adhering strictly to the manufacturer’s instructions. We then meticulously counted the TUNEL-positive neurons surrounding the hematoma in each brain using the ImageJ software. The TUNEL-positive neuron counts were reported as a percentage (%).

### Reduced glutathione/oxidized glutathione disulfide ratio

The drug concentrations and plasmid transfection methods for PC12/SH-SY5Y cells (2 × 10^5^ cells) adhered to the protocols outlined in the “*In vitro* Model of ICH and Treatments” The experimental grouping was designed with reference to Flow Cytometry, while the *vivo* models and their respective groupings were based on the protocols described in the animal experimentation section. As directed by the kit’s instructions, tissue samples were stored in liquid nitrogen and analyzed by weighing equal masses of tissue. Fresh cells were pretreated following the provided guidelines. The ratios of reduced glutathione (GSH) to oxidized glutathione disulfide (GSSG) in both cellular and brain tissue samples were determined using a GSH and GSSG Assay Kit (Beyotime Biological Technology Co., China). Adhering strictly to standard protocols, positive, negative, and blank controls were carefully established in accordance with the instructions. A standard curve was plotted from the standard test results, with corresponding calculations executed. The results were normalized and presented as the GSH/GSSG ratio, expressed as fold change.

### Measurement of PGE2 concentration

The drug concentrations and plasmid transfection methods for PC12/SH-SY5Y cells (2 × 10^5^ cells) adhered to the protocols outlined in the “*In vitro* Model of ICH and Treatments”. The experimental grouping was designed with reference to Flow Cytometry, while the *vivo* models and their respective groupings were based on the protocols described in the animal experimentation section. For cell samples, supernatants were collected, while for animal samples, serum was collected and diluted 200-fold for normalization. The production of PGE2 was measured using a PGE2 ELISA kit (EK8103/2, Lianke Biotech, China), following the kit’s instructions. The 96-well plate was inserted into a microplate reader where OD readings were taken at 450 nm (absorption) and 630 nm (reference). The standard curve ranged from 15.63 to 2,000 pg/mL, and the results for PGE2 were expressed in pg/mL.

### Cell transfection

COX2 overexpression plasmid/overexpression negative control (NC) (2 μg,pcDNA3.1,Nanjing Juxiong Biological Co., China). PC12 or SH-SY5Y cells were plated in 6-well dishes at a concentration of 200,000 cells per well. Once the cultures hit 70% confluency, they underwent transfection with either the COX2 overexpression construct or the negative control plasmid, employing Lipofectamine 3,000 (Thermo Fisher Scientific) as per the supplier’s guidelines. After 6 h of transfection, the medium was replaced with complete medium, and the cells were cultured for another 24 h. Proteins were extracted from the cells after transfection, and Western blot analysis was performed to detect COX2 expression and evaluate transfection efficiency (n = 3). The transfected cells were utilized to evaluate their potential in reducing the pharmacological effects of LCD.

### Statistical analysis

This study selects the total sample size or group size based on literature review. During the experiment, the researchers applied a blinded approach to the allocation of treatments and the evaluation of outcomes. All animals and data points were included in the analysis. In the research design involving vertebrates and cell lines, gender is not considered as a factor. Initially, we assigned five rats per group to account for possible losses. Ultimately, six rats that failed to exhibit significant hematoma were excluded from the study, as they did not display neurological deficits post-surgery. (Post-euthanasia examination revealed the absence of hematoma in their brains). This exclusion ensured a final sample size of three rats per group (n = 3). All experiments were biologically replicated multiple times, as indicated in the figure legends. Statistical analyses were performed using GraphPad Prism 8. Samples were compared using one-way ANOVA. Since the data exhibited unequal variances, data transformation was performed to achieve homogeneity of variances. After confirming the homogeneity of variances, Tukey’s test was conducted to compare the means of the four groups. *: *p* < 0.05; **: *p* < 0.01, n. s.: not statistically significant.

## Results

### LCD inhibits neuronal cell death and oxidative stress levels under ischemic and hypoxic conditions *in vitro*

**FIGURE 1:**
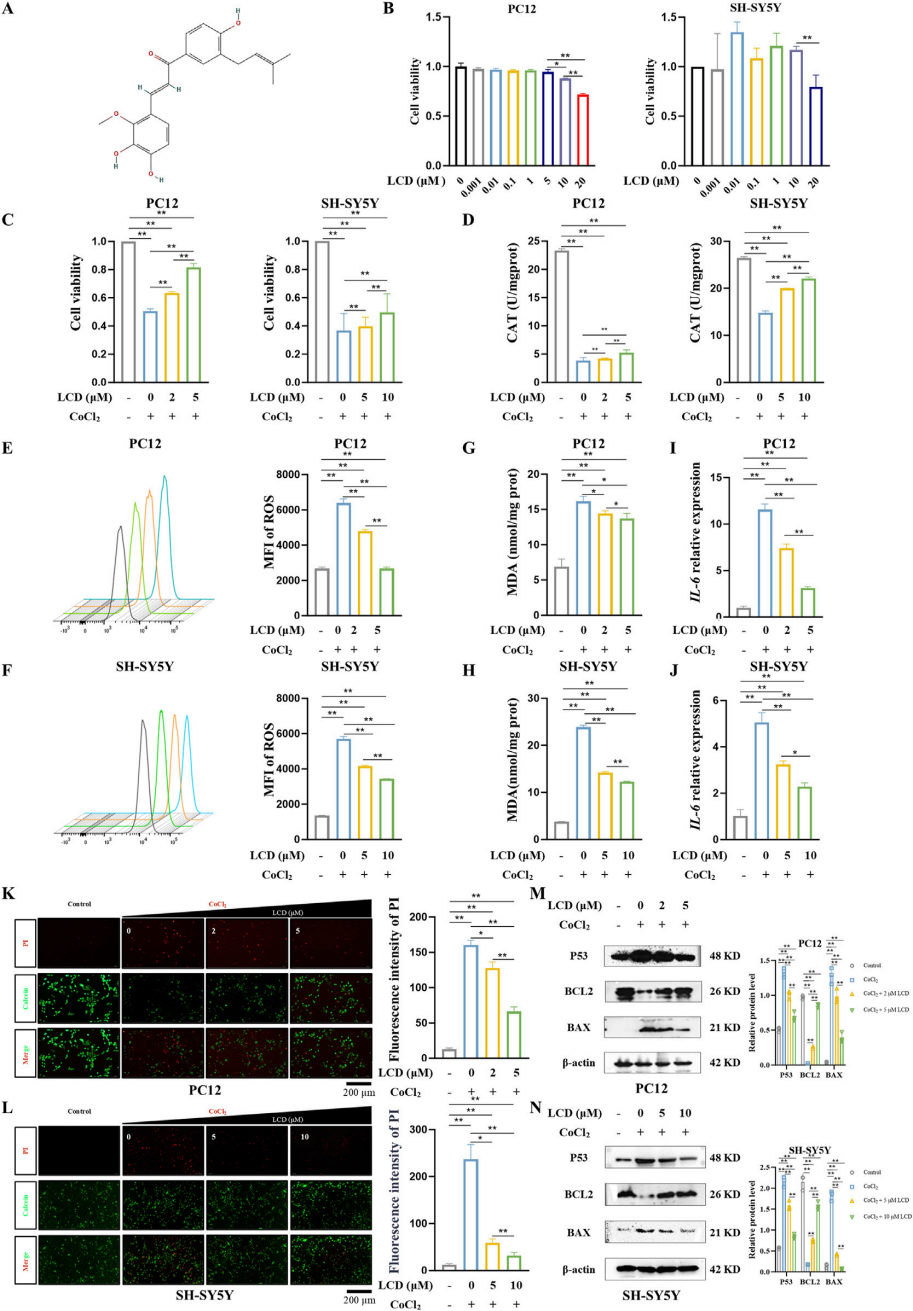
LCD Mitigates CoCl_2_-Induced Neuronal Cell Death and Reduces Oxidative Stress Levels. **(A)** Presentation of the structural formula for LCD. **(B)** PC12 and SH-SY5Y cells were treated with different LCD doses for 24 h (0.001–20 µM. Cell viability post-exposure was evaluated using the CCK-8 assay; results are presented as mean ± SD (n = 3). **(C)** PC12 or SH-SY5Y cells were pretreated with LCD (0, 2/5 µM or 0, 5/10 µM) for 2 h, then exposed to CoCl_2_ (400 μg/mL) for 24 h. Cell viability was measured using the CCK-8 assay, with results expressed as mean ± SD (n = 3). **(D)** PC12 or SH-SY5Y cells were subjected to the same treatment regimen as in **(C)**. After the 24 h CoCl_2_ treatment, the level of CAT was measured using the CAT assay. Data are expressed as mean ± SD (n = 3). **(E–F)** PC12 or SH-SY5Y cells underwent LCD pretreatment as in **(C)** and subsequently treated with CoCl_2_. After 24 h, the MFI of ROS was assessed using flow cytometry. The data are presented as mean ± SD (n = 3). **(G–H)** PC12 or SH-SY5Y cells were treated as described in **(C)**, and after the 24 h CoCl_2_ treatment, the levels of MDA were quantified via the MDA assay, with results expressed as mean ± SD (n = 3). **(I–J)** PC12 or SH-SY5Y cells underwent LCD pretreatment as in **(C)** and subsequently treated with CoCl_2_. Following the 24 h treatment, *IL-6* mRNA expression was quantified by qRT-PCR, with results expressed as mean ± SD (n = 3). **(K–L)** PC12 or SH-SY5Y cells were exposed to LCD and CoCl_2_ as per protocol **(C)**. After 24 h, cell death was quantified using the Live/Dead assay (Calcein-AM/PI staining), with the proportion of dead cells expressed as mean ± SD (n = 3). Scale bar = 200 μm. **(M–N)** PC12 or SH-SY5Y cells were subjected to the same treatment regimen as in **(C)**. After the 24 h CoCl_2_ treatment, the levels of P53, BCL2, and BAX proteins were quantified by Western blot, with β-actin as the loading control. Data are presented as mean ± SD (n = 3). Statistical significance: **p < 0.01, *p < 0.05.

The potential toxicity of LCD (Figure 1A) was evaluated in rat and human neuronal cells (PC12 and SH-SY5Y) at various concentrations over a 24 h period. LCD demonstrated excellent biological safety, exerting no impact on cell activity at concentrations up to 5 μM in PC12 cells and 10 μM in SH-SY5Y cells (Figure 1B). To assess LCD’s neuroprotective impacts on SBI following ICH, an ischemic and hypoxic environment was mimicked using CoCl_2_. Notably, LCD treatment significantly enhanced cell viability (Figure 1C. PC12 cells: 1.6-fold increase with 5 μM LCD treatment; SH-SY5Y cells: 1.3-fold increase with 10 μM LCD treatment). CAT, a key endogenous antioxidant, significantly decreased under CoCl_2_-induced oxidative stress. LCD inhibited this depletion of CAT (Figure 1D. PC12 cells: 1.4-fold increase with 5 μM LCD treatment; SH-SY5Y cells: 1.5-fold increase with 10 μM LCD treatment). Furthermore, LCD-treated PC12 and SH-SY5Y cells scavenged ROS (Figures 1E,F. PC12 cells: 2.4-fold decrease with 5 μM LCD treatment; SH-SY5Y cells:1.7-fold decrease with 10 μM LCD treatment) and reduced MDA levels (Figures 1G,H. PC12 cells: 1.2-fold decrease with 5 μM LCD treatment; SH-SY5Y cells: 1.9-fold decrease with 10 μM LCD treatment) generated by CoCl_2_-induced oxidative stress. Additionally, evidence indicates that exposure to CoCl_2_ triggers an inflammatory response, marked by increased *IL-6* secretion (Montopoli et al., 2007; Schmalz et al., 2000). Remarkably, LCD-treated PC12 and SH-SY5Y cells mitigated *IL-6* secretion (Figures 1I,J. PC12 cells: 3.7-fold decrease with 5 μM LCD treatment; SH-SY5Y cells:2.2-fold decrease with 10 μM LCD treatment). Live/dead assays showed LCD reduced hypoxia-induced neuronal death in a dose-dependent manner (Figures 1K,L. PC12 cells: 2.4-fold decrease with 5 μM LCD treatment; SH-SY5Y cells: 7.5-fold decrease with 10 μM LCD treatment). The protein expression analysis unveiled a marked decrease in cell death markers P53 and BAX, paralleled by a substantial increase in the anti-apoptotic protein BCL-2. Consequently, this observation offers compelling evidence that LCD possesses a remarkable capability to mitigate cell death under hypoxic conditions (Figures 1M,N).

### LCD directly targets COX2 to participate in lipid peroxidation

**FIGURE 2:**
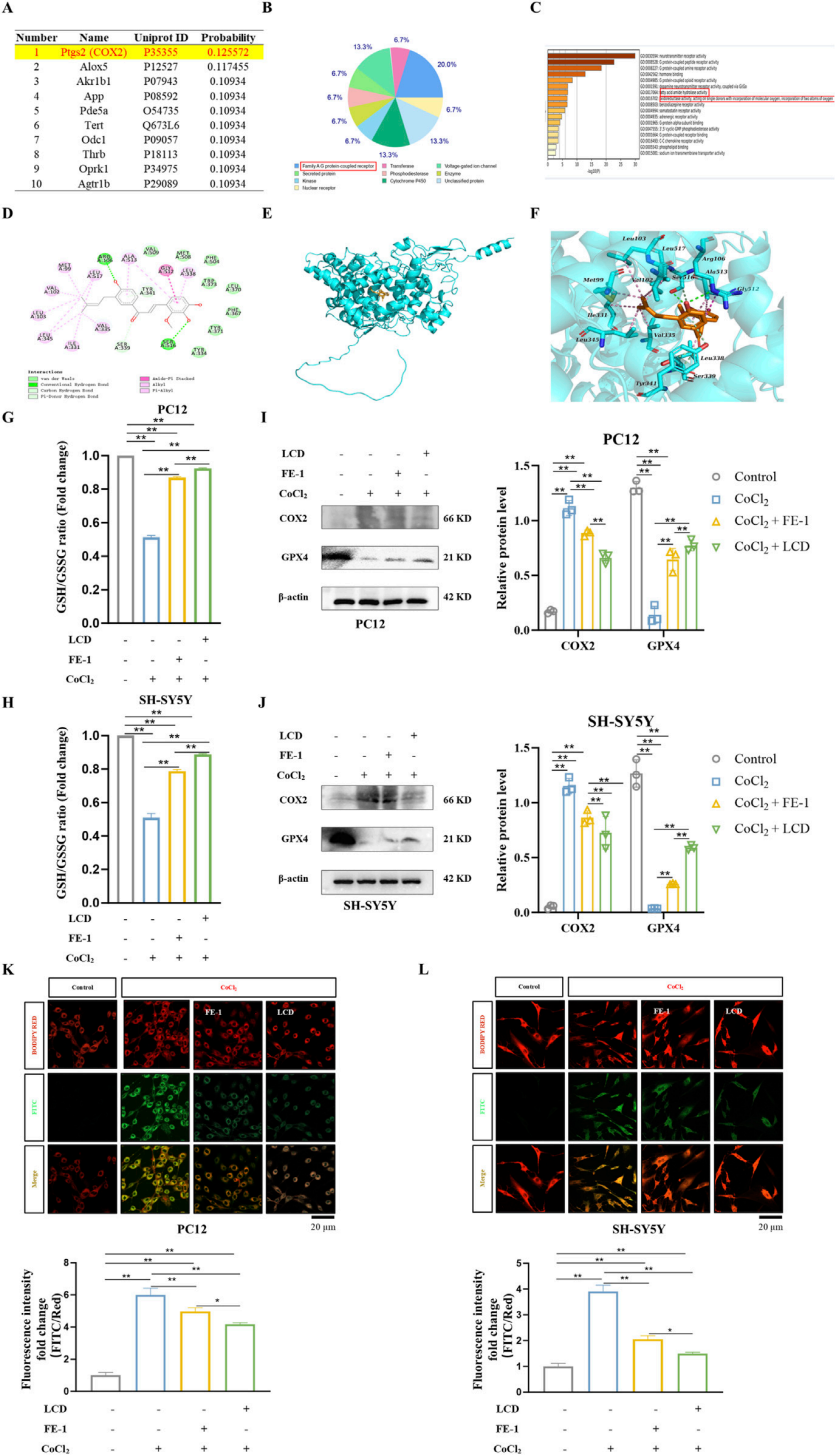
LCD Acts as a Ferroptosis Inhibitor in ICH Neuronal Cells. **(A)** The top ten genes with direct binding correlation to LCD, as predicted by SWISS TARGET DATA. **(B)** A pie chart categorizing the genes predicted by SWISS TARGET DATA to have direct binding correlation with LCD (102 genes, *p* < 0.05). **(C)** GO term analysis providing insights into the LCD target. **(D–F)** The 2D binding mode of LCD with rat COX2 is illustrated in **(D-F)** presents the 3D binding mode of LCD with rat COX2. LCD is highlighted in orange, while the backbone of rat COX2 is represented by a cyan surface and cartoon rendering. The residues within the binding pocket of rat COX2 are displayed as cyan sticks. Conventional hydrogen bond interactions are indicated by green dashed lines, carbon-hydrogen bond interactions by pale green dashed lines, alkyl-alkyl hydrophobic interactions by pink dashed lines, and amide-pi stacked hydrophobic interactions by light magenta dashed lines. **(G–H)** PC12 or SH-SY5Y cells were subjected to treatment with LCD (5 or 10 µM) or FE-1 (1 µM) for 2 h, followed by exposure to CoCl_2_ (400 μg/mL) for an additional 24 h. The ratio of GSH/GSSG was subsequently quantified using a dedicated GSH/GSSG assay. Data are presented as mean ± SD (n = 3). **(I–J)** Following treatment of PC12 or SH-SY5Y cells with LCD (5 or 10 µM) or FE-1 (1 µM) for 2 h, and subsequent exposure to CoCl_2_ (400 μg/mL) for 24 h, COX2 and GPX4 protein levels were quantified by Western blot, using β-actin as the loading control. Data are presented as mean ± SD (n = 3). **(K–L)** PC12 or SH-SY5Y cells were pretreated with LCD (5 or 10 µM) or FE-1 (1 µM) for 2 h, followed by CoCl_2_ (400 μg/mL) exposure for an additional 24 h. Oxidized and reduced dye fluorescence intensities were measured using FITC (green) and BODIPY RED (red), respectively. The ratio of FITC/BODIPY RED fluorescence intensities served as a readout for cellular lipid peroxidation. Fluorescence density quantifications were normalized prior to plotting. Scale bars = 20 μm. Data are presented as mean ± SD (n = 3). Statistical significance is denoted by **for *p* < 0.01 and * for *p* < 0.05.

Previously, it was shown that LCD alleviates SBI following ICH. To uncover the molecular mechanism underlying LCD’s impact, SWISS-TARGET was utilized to predict potential targets, and COX2 was identified as the most highly ranked, concurrently serving as a biomarker generated during the process of ferroptosis (Figures 2A,B). Lipid peroxidation, a vital mechanism recognized for initiating ferroptosis, initiates with the oxidation of arachidonic acid (AA) through the action of lipoxygenases (LOX), as well as free radicals and iron, culminating in the generation of the AA-OOH-PE complex (Bai et al., 2019). Alternatively, AA can be catalyzed by cyclooxygenases (COX) to produce prostaglandins, which are further converted into lipid peroxides involved in ferroptosis (Li et al., 2022). GO enrichment analysis revealed that the targets of LCD are mainly enriched in neurotransmitter receptors, fatty acid metabolism, and oxidoreductase activity, which is consistent with the pathological response of lipid peroxidation in ferroptosis (Figure 2C). Thus, it is hypothesized that COX2 is targeted by LCD to influencing in the ferroptosis process of ICH neuronal cells.

For this purpose, molecular docking predictions were conducted for the binding site between LCD and COX2 (Figures 2D–F). The docking score of LCD with rat COX2 was −8.36 kcal/mol. A lower docking score reflects higher ligand-receptor binding strength. The predicted binding mode of LCD and rat COX2 was illustrated. LCD formed a suitable steric complementarity with the binding site of rat COX2. LCD and rat COX2 exhibited conventional hydrogen bonding, carbon-hydrogen bonding, hydrophobic associations, and Van der Waals (VDW) interactions. Specifically, the oxygen atoms of LCD formed two conventional hydrogen bond interactions with the residues of Ser516 and Arg106 in rat COX2, three carbon hydrogen bond interactions with the residues of Ser339 and Ser516, and one pi-donor hydrogen bond interaction with the residue of Tyr341. The carbon atoms of LCD formed alkyl-alkyl hydrophobic contacts with Val335, Ile331, Leu345, Leu517, Met99, Val102, and Leu103. LCD formed amide-pi stacked hydrophobic interactions with the residues of Gly512 and Ala513, and pi-alkyl hydrophobic interactions with the residues of Leu338 and Ala513. VDW interactions were formed between LCD and the residues of Val509, Leu370, Met508, Phe504, Trp373, Phe367, Tyr371, and Tyr334 in rat COX2. These interactions primarily drove the binding affinity of LCD for rat COX2.

### LCD improves ferroptosis of neuronal cells after ICH by acting as a ferroptosis inhibitor

Ischemia-hypoxia models were induced in PC12 and SH-SY5Y cells using CoCl_2_, and changes in GSH/GSSG ratio, ferroptosis-regulating proteins (COX2, GPX4), mitochondrial lipid ROS levels, and lipid peroxidation were observed. These indicators are crucial in the ferroptosis process post-ICH (Yan et al., 2020). FE-1, a known ferroptosis inhibitor, served as a control. It was found that ischemia-hypoxia decreased the GSH/GSSG ratio and GPX4 levels, while increasing COX2 levels and mitochondrial lipid ROS. Intervention with LCD and FE-1 restored the GSH/GSSG ratio (Figures 2G,H. PC12 cells: 1.8-fold increase with LCD treatment and 1.7-fold increase with FE-1 treatment; SH-SY5Y cells: 1.7-fold increase with LCD treatment and 1.5-fold increase with FE-1 treatment) and GPX4 (Figures 2I,J) activity, and reversed COX2 expression and ROS levels (Figures 2K,L. PC12 cells: 1.4-fold decrease with LCD treatment and 1.2-fold decrease with FE-1 treatment; SH-SY5Y cells: 2.6-fold decrease with LCD treatment and 1.9-fold decrease with FE-1 treatment). The results above have demonstrated that the same pharmacological effects can be produced by LCD as by FE-1. Consequently, it is believed that ferroptosis in neuronal cells can be reduced by LCD, which can serve as a ferroptosis inhibitor, during SBI after ICH.

### Overexpression of COX2 eliminates the inhibitory impact of LCD on oxidative stress in neuronal cells during SBI after ICH

**FIGURE 3:**
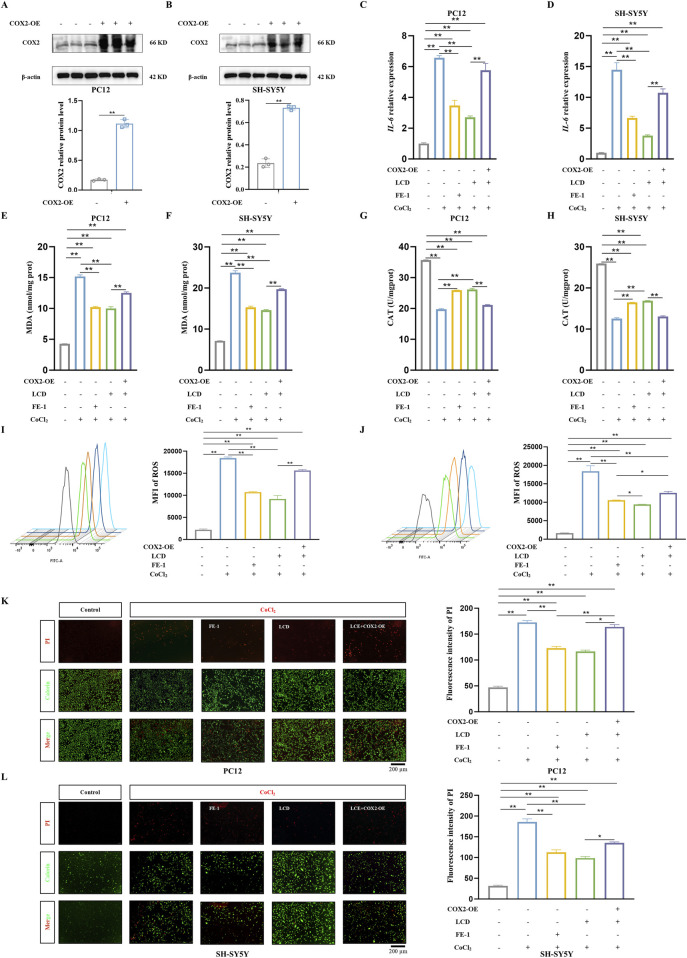
Overexpression of COX2 Blocks the Improvement of Oxidative Stress and Cell Death in ICH Neuronal Cells by LCD. **(A–B)** PC12 or SH-SY5Y cells were transfected with the COX2-overexpressing (COX2-OE) plasmid for 48 h, followed by quantification of COX2 protein levels via Western blot using β-actin as the loading control. Data are presented as mean ± SD (n = 3). **(C–D)** PC12 or SH-SY5Y cells, transfected with or without the COX2-OE plasmid for 24 h, were then exposed to LCD (5 or 10 µM) or FE-1 (1 µM) for 2 h. This treatment was followed by a 24 h incubation with CoCl_2_ (400 μg/mL). Following the 24-h incubation, *IL-6* mRNA expression was quantified by qRT-PCR. Data are expressed as mean ± SD (n = 3). **(E–F)** PC12 or SH-SY5Y cells, underwent the same treatment protocol as described in **(C)**. Following this treatment, the levels of MDA were assessed using an MDA assay. Data are expressed as mean ± SD (n = 3). **(G–H)** PC12 or SH-SY5Y cells were subjected to the identical treatment regimen as in **(C)**. After the 24 h treatment, the level of CAT was evaluated using a CAT assay. Data are expressed as mean ± SD (n = 3). **(I–J)** PC12 or SH-SY5Y cells were exposed to the identical treatment protocol as described in **(C)**. After 24 h, the MFI of ROS was quantified using flow cytometry. Data are expressed as mean ± SD (n = 3). **(K–L)**. PC12 or SH-SY5Y cells were subjected to the identical experimental protocol as outlined in **(C)**. Following the 24 h treatment, cell death was assessed using the Live (Calcein, *green*)/Dead (PI, *red*) assay. The percentage of dead cells was determined based on the Live/Dead staining, and the results are presented as mean ± SD (n = 3). Scale bar = 200 μm. Statistical significance: **p < 0.01, *p < 0.05.

To provide further substantiation that LCD specifically targets and inhibits COX2, thereby mitigating secondary injury after ICH, we utilized a COX2-overexpression (COX2-OE) plasmid to induce COX2 overexpression in neuronal cells (PC12 and SH-SY5Y cells) (Figures 3A,B). Upon examining oxidative stress markers, the results clearly showed that COX2’s excess activity negated LCD’s suppressive influence on the inflammatory cytokine *IL-6*, when juxtaposed with the group treated exclusively with LCD (Figures 3C,D. PC12 cells: 2.1-fold increase with COX2 overexpression, SH-SY5Y cells: 2.8-fold increase with COX2 overexpression). Furthermore, in cells overexpressing COX2, LCD treatment resulted in heightened levels of MDA (Figures 3E,F. PC12 cells: 1.3-fold increase with COX2 overexpression, SH-SY5Y cells: 1.3-fold increase with COX2 overexpression) and ROS (Figures 3I–J. PC12 cells: 1.7-fold increase with COX2 overexpression, SH-SY5Y cells: 1.3-fold increase with COX2 overexpression) compared to the group administered LCD alone, while the CAT levels were markedly decreased (Figures 3G,H. PC12 cells: 0.8-fold decrease with COX2 overexpression, SH-SY5Y cells: 0.7-fold decrease with COX2 overexpression). Additionally, an assessment of the ratio of dead to viable cells in PC12 and SH-SY5Y cultures revealed that COX2 overexpression reduced LCD’s neuroprotective impact against cell death in the ICH model (Figures 3K,L. PC12 cells: 1.4-fold increase with COX2 overexpression, SH-SY5Y cells: 1.4-fold increase with COX2 overexpression). The results indicate that LCD enhances neuronal cell inflammatory responses, ROS levels, and ferroptosis by inhibiting COX2.

### LCD ameliorates ferroptosis in neuronal cells of *in vitro* ICH by inhibiting the COX2-targeted PGE2/EP1 pathway

**FIGURE 4:**
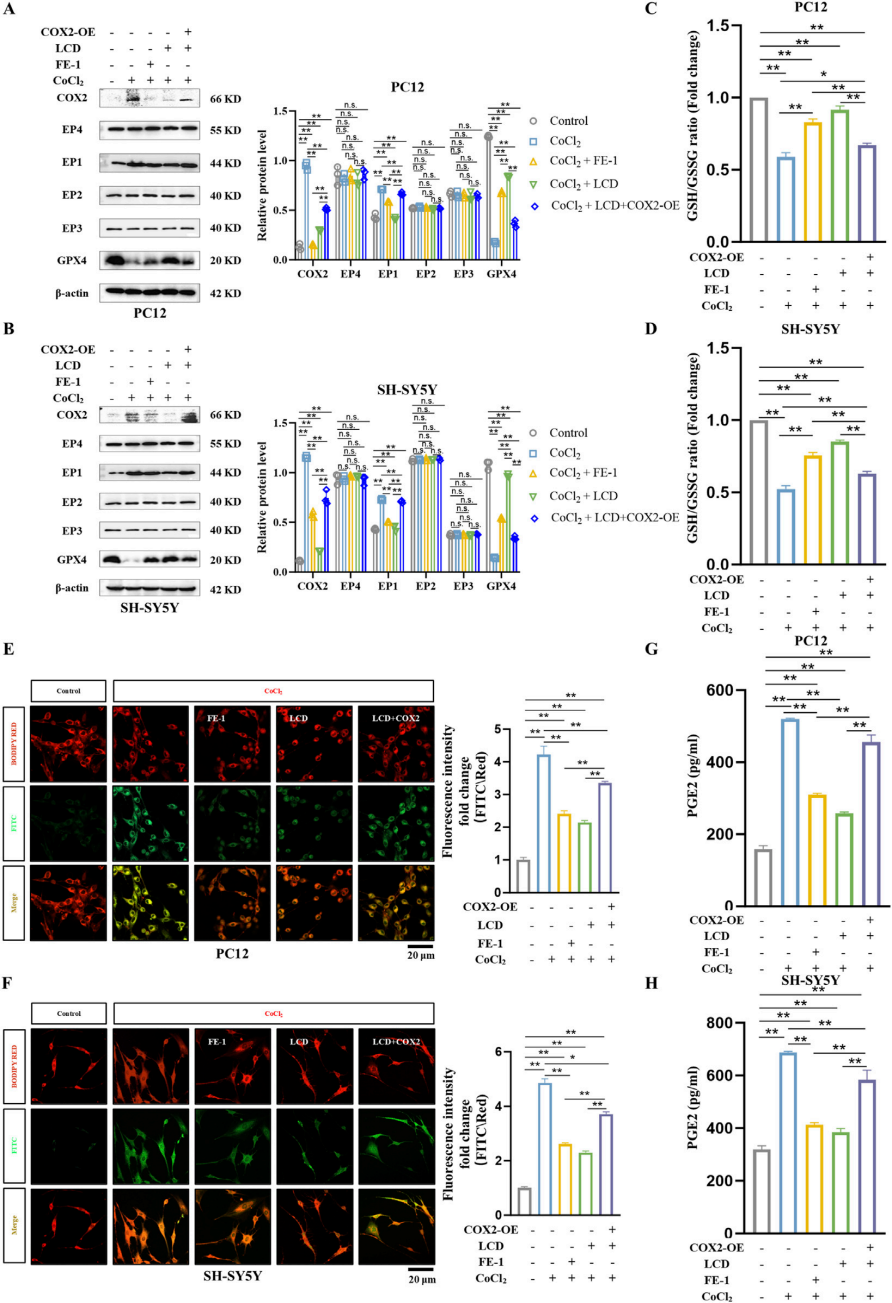
LCD Improves Ferroptosis in ICH Neuronal Cells *In Vitro* by Targeting the COX2/PGE2/EP1 Pathway. **(A–B)** PC12 or SH-SY5Y cells were transfected with a COX2-overexpressing (COX2-OE) plasmid or a control vector for 24 h, followed by exposure to LCD (5 or 10 µM) or FE-1 (1 µM) for 2 h. This was followed by a 24 h incubation with CoCl_2_ (400 μg/mL). After the incubation period, protein levels of COX2, EP1-4, and GPX4 were quantified by Western blot, using β-Actin as the loading control. Data are presented as mean ± SD (n = 3). **(C–D)** PC12 or SH-SY5Y cells were subjected to the same treatment as in (A). After 24 h incubation with CoCl_2_, the ratio of GSH/GSSG was measured using a dedicated GSH/GSSG quantification assay. Data are presented as mean ± SD (n = 3). **(E–F)** PC12 or SH-SY5Y cells underwent the same treatment regimen as described in (A). Following the 24 h incubation with CoCl_2_, mitochondrial lipid ROS levels were quantified using BODIPY RED fluorescence. The oxidized dye was measured using FITC fluorescence (*green*), the reduced dye was quantified using BODIPY RED fluorescence (*red*). The ratio of the fluorescence intensities at FITC and BODIPY RED channels was calculated to assess lipid peroxidation in cells. Quantification of fluorescence density were normalized before plotting. Scale bars are provided 20 μm. Bars represent the mean ± SD (n = 3). **(G–H)** PC12 or SH-SY5Y cells were subjected to the identical treatment regimen as in **(A)**. After the 24 h incubation period, the protein level of PGE2 was measured using an ELISA assay. Data are presented as mean ± SD (n = 3). Statistical significance is indicated as follows: **p < 0.01, *p < 0.05, and n. s.; not significant.

Current research widely acknowledges that excessive oxidative stress and dysfunction of the cellular antioxidant system are the primary drivers of ferroptosis (Guo et al., 2019). Building on this foundation, we examined the pharmacological effects of LCD on neural cell ferroptosis in the CoCl_2_ model, specifically under conditions where COX2 is overexpressed. Our research indicates that during LCD therapy with COX2 upregulation in neural cells, we observe a significant suppression of the anti-ferroptotic protein GPX4’s function (Figures 4A,B) and a moderate reduction in the GSH/GSSG balance (Figures 4C,D. PC12 cells: 0.7-fold decrease with COX2 overexpression, SH-SY5Y cells: 1.4-fold decrease with COX2 overexpression). The inhibitory impacts of LCD on lipid peroxide levels were entirely abrogated by COX2 overexpression (Figures 4E,F. PC12 cells: 1.6-fold increase with COX2 overexpression, SH-SY5Y cells: 1.6-fold increase with COX2 overexpression).

Studies have shown that prostaglandin PGE2 is a major metabolite of COX2 enzyme activity in the brain (Sluter et al., 2023). Therefore, we quantified PGE2 expression using ELISA and found that PGE2 expression decreased upon LCD intervention in the CoCl_2_ model but recovered after COX2 overexpression (Figures 4G,H. PC12 cells: 1.7-fold increase with COX2 overexpression, SH-SY5Y cells: 1.5-fold increase with COX2 overexpression). Additionally, we observed EP1-EP4 receptor levels at the protein level and found that EP1 expression changed in response to COX2 expression (Figures 4A,B). Thus, we conclude that LCD potentially ameliorates ferroptosis in neuronal cells of ICH by inhibiting the COX2-targeted PGE2/EP1 pathway.

### LCD significantly ameliorates SBI after ICH in rats

**FIGURE 5:**
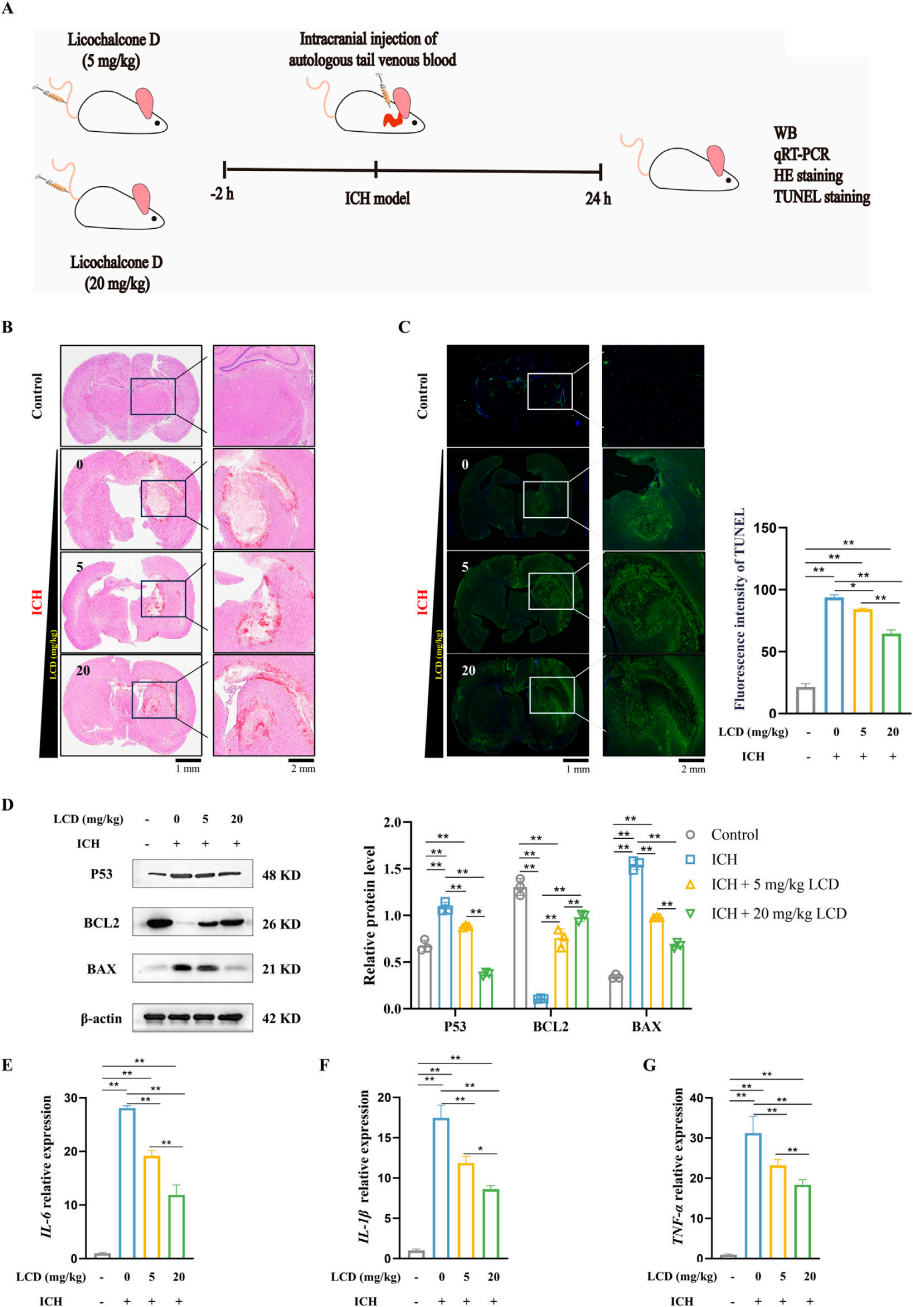
LCD Ameliorates Neuronal Cell Death and Oxidative Stress Levels in SBI After Rat ICH. **(A)** Experimental schedule/regimen. Rats administered LCD were randomly assigned to four treatment groups: 1) Control; 2) ICH group; 3) ICH + LCD 5 mg/kg group; 4) ICH + LCD 20 mg/kg group. These treatments were administered to the rat via tail intravenous (i.v.) injection. **(B)** Coronal sections of brain tissue were stained with HE to assess intracranial hematoma. Scale bars are provided at 1 mm or 2 mm. **(C)** Coronal brain sections were subjected to TUNEL fluorescent staining (*green*). With DAPI (blue) used for nuclear counterstaining. Scale bars: 1 mm (low-magnification images) or 200 μm (high-magnification insets). Data are presented as mean ± SD (n = 3). **(D)** Western blot analysis was performed to quantify the relative protein expression of P53, BAX, and BCL2 in brain tissues. β-Actin was used as an internal loading control. Data are presented as mean ± SD (n = 3). **(E–G)** qRT-PCR was employed to assess the mRNA levels of *IL-6, IL-1β*, and *TNF-α* in brain tissues. Expression levels were normalized to the housekeeping gene *β-Actin* prior to statistical analysis. Data are expressed as mean ± SD (n = 3). Statistical significance is denoted as: **p < 0.01, *p < 0.05.

To investigate LCD’s impact on ICH, we developed a rat model of spontaneous ICH and determined the therapeutic concentration based on previous reports (Zhang et al., 2024b) (Figure 5A). Rats were pretreated with LCD via tail vein injection. H&E staining 24 h post-ICH showed neuron loss, interstitial edema, and red blood cell infiltration at the hematoma site, with a modest reduction in hematoma area with LCD (Figure 5B). TUNEL staining revealed decreased neuronal cell death upon LCD administration (Figure 5C. 1.4-fold decrease with 20 mg/kg LCD treatment). Western blot revealed decreased P53 and BAX expression along with increased BCL2 levels. Supporting the notion that LCD can mitigate cell death resulting from ICH (Figure 5D). Additionally, LCD reduced inflammatory cytokine mRNA (*IL-6, IL-1β, TNF-α*) in brain tissue (Figures 5E–G. 2.4, 2.0 or 1.7-fold decrease with 20 mg/kg LCD treatment).

### LCD Improves Ferroptosis in Rat ICH by inhibiting the COX2-Targeted PGE2/EP1 pathway

**FIGURE 6:**
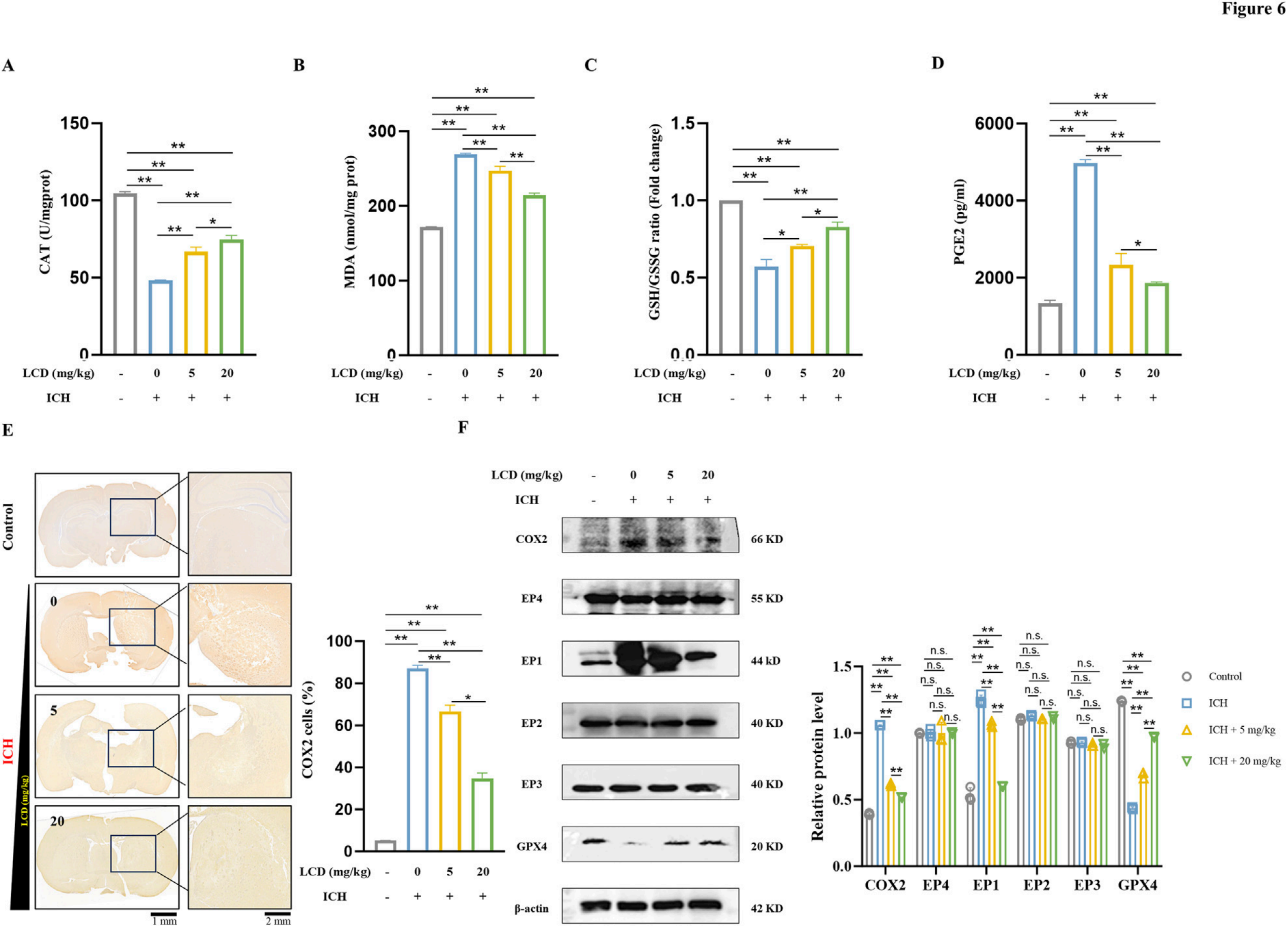
LCD Improves Ferroptosis in Rat ICH by Inhibiting the COX2/PGE2/EP1 Pathway. **(A)** The CAT activity in brain tissues was were quantified using a CAT assay and normalized prior to plotting. Data are presented as mean ± SD (n = 3). **(B)** The levels of MDA in brain tissue were measured using an MDA assay and normalized before plotting. Data are expressed as mean ± SD (n = 3). **(C)** The ratio of GSH/GSSG in brain tissue was quantified using a GSH/GSSG assay and normalized prior to plotting. Data are expressed as mean ± SD (n = 3). **(D)** The levels of PGE2 in brain tissue were assessed using a PGE2 ELISA assay and normalized before plotting. Data are expressed as mean ± SD (n = 3). **(E)** Immunohistochemical staining of COX2 was performed on coronal sections of brain tissue, and the results were normalized before plotting. Data are expressed as mean ± SD (n = 3). Scale bars of 1 mm or 2 mm are provided. **(F)** Western blot analysis was conducted to quantify the relative protein expression of COX2, EP1-EP4, and GPX4 proteins in brain tissue, β-Actin was used as an internal loading control. Error bars represent standard deviation and data are expressed as the mean ± SD, n = 3. Statistical significance is denoted as: **p < 0.01, *p < 0.05 and n. s., no significant difference.

The mechanism of action of LCD in rat ICH was investigated, particularly its potential to attenuate ferroptosis by inhibiting the COX2/PGE2/EP1 pathway. Brain tissue oxidative stress levels were initially evaluated. It was found that the antioxidant enzyme CAT was significantly reduced in the ICH model and gradually increased with the introduction of LCD treatment (Figure 6A. 1.6- fold increase with 20 mg/kg LCD treatment). Concurrently, MDA levels decreased with LCD treatment, indicating oxidative stress in ICH and LCD’s mitigating impact (Figure 6B. 1.3-fold decrease with 20 mg/kg LCD treatment). Subsequently, indicators related to ferroptosis were examined. A decrease in the GSH/GSSG ratio (Figure 6C. 1.4-fold increase with 20 mg/kg LCD treatment) and GPX4 (Figure 6F) protein levels was observed after the onset of cerebral hemorrhage, which were restored following LCD treatment. This result emphasized that LCD can mitigate lipid peroxidation during ferroptosis in rat ICH. To further substantiate LCD’s role in the ferroptosis pathway in rat ICH, IHC analysis of COX2 was performed on coronal sections of rats receiving gradient doses of LCD, and COX2 expression was evaluated at the protein level. It was demonstrated that COX2 expression was elevated in the rat autologous blood ICH model group and gradually decreased with LCD intervention (Figure 6E. 2.5-fold decrease with 20 mg/kg LCD treatment; Figure 6F). Using the ELISA method, PGE2 expression in brain tissue was quantified, it was found that, as a metabolite of COX2, PGE2 levels also declined with the reduction in COX2 (Figure 6D. 2.7-fold decrease with 20 mg/kg LCD treatment). Finally, the expression of EP1-EP4 protein levels in brain tissue was examined, and it was found that EP1 expression decreased as COX2/PGE2 levels diminished (Figure 6F).

## Discussion

This research first revealed LCD’s neuroprotective impact in a rat ICH model and investigated its mechanisms. It has been reported in multiple literature sources that small lipophilic compounds, with fewer than 8 hydrogen bonds and a molecular weight of less than 400 Da, are capable of crossing the blood-brain barrier (BBB) (Choudhari et al., 2021). LCD with a molecular weight of 354.4 and 3 hydrogen bonds, meets the criteria for BBB penetration. Literature corroborates that LCD and LCB, key bioactive chalcones isolated from *G. inflata Batalin [Fabaceae]*, neutralize ROS (Haraguchi et al., 1998). Furthermore, LCB ameliorates stroke by suppressing oxidative stress via Nrf2 signaling (Zhou et al., 2021). Thus, it is hypothesized that LCD crosses the BBB to attenuate SBI in ICH via redox modulation and anti-inflammatory signaling. Previous studies have explored LCD’s pharmacological effects in human melanoma via oral gavage (Si et al., 2018). LCD, a sparingly water-soluble lipophilic flavonoid, exhibits impaired gastrointestinal absorption due to its suboptimal dissolution kinetics in physiological fluids. Given the complexities of oral drug absorption, we decided to employ a different route of administration in our rat ICH model. Specifically, LCD was administered via the tail vein as a pretreatment, 2 hours before ICH induction, to facilitate a more precise evaluation of its pharmacological effects. The study highlights LCD’s potential pharmacological effects in mitigating symptoms of SBI linked to ICH.

Recent investigations reveal that secondary neuronal death after ICH involves multiple regulated cell death pathways, with ferroptosis playing a key role alongside toxic environments and inflammation. A study (Li et al., 2017) demonstrated that the ferroptosis inhibitor Ferrostatin-1 (FE-1) decreased neuronal necrosis and improved neurological results in an intracerebral hemorrhage rodent model. Cells treated with FE-1 were administered according to the manufacturer’s protocols and functioned as experimental controls during LCD testing. The study revealed that LCD mitigated SBI by inhibiting neuronal ferroptosis triggered by lipid peroxidation, and its pharmacological effects were comparable to those achieved by FE-1 treatment.

The literature (Zhang et al., 2022) indicates that excessive iron production in SBI post-ICH instigates oxidative stress through the Fenton reaction, resulting in ROS formation. Iron chelators have been shown to mitigate iron accumulation and subsequent ROS production, thereby conferring additional anti-inflammatory benefits. These benefits encompass the modulation of inflammatory factor release and the enhancement of neurological function. Therefore, the pharmacological effects of LCD pretreatment in alleviating oxidative stress and inflammation following ICH were explored in our study. The results demonstrate that LCD plays a crucial neuroprotective role in mitigating SBI by suppressing ferroptosis, reducing oxidative stress, and curbing the release of inflammatory mediators in neuronal cells.

In the rat brain, neuronal COX2, a metabolite associated with ferroptosis, plays crucial roles in essential physiological functions such as synaptic transmission (Breder et al., 1995; Jung et al., 2019), and neuropeptide release (Yin et al., 2022). In our study, we discovered that LCD binds to COX2. Additionally, we observed a significant discrepancy between the predicted probability of COX2 interaction (0.125) and the high affinity suggested by the docking score (−8.36 kcal/mol). The low predicted probability may be attributed to limitations in the database or the model’s oversimplification of specific binding mechanisms, while the high docking score directly reflects the spatial compatibility between LCD and the active site of COX2. This discrepancy highlights the fact that relying solely on one method may not fully capture the complexities of molecular interactions. Meanwhile, small-molecule metabolites like chalcones, characterized by their polyphenolic nature, tend to exhibit non-specific “docking” effects. In future research, molecular dynamics simulations will be further utilized to score potential binding sites and exclude non-specific targets through benchwork experiments. Subsequently, the model parameters can be refined to more accurately assess the affinity between LCD and COX2.

Subsequently, further exploration into the signaling pathways mediating the role of LCD-induced COX2 in SBI was conducted using both *in vitro* and *in vivo* ICH models. A potential molecular mechanism is being investigated, through which LCD alleviates SBI by blocking the COX2/PGE2/EP1 signaling pathway, thereby curbing mitochondrial lipid peroxidation and halting the progression of neuronal ferroptosis. However, the pharmacological effects of LCD were not completely reversed by the overexpression of COX2 (COX2-OE), indicating that additional binding sites for LCD may potentially exist. It has been demonstrated by previous studies that oxidative stress-induced aging can be ameliorated by LCD through the AMPK pathway (Maharajan et al., 2021). Additionally, it has been shown that neuroinflammation and neuronal apoptosis following ICH can be mitigated by Irisin through the αVβ5 integrin/AMPK signaling axis (Wang et al., 2022). It was also disclosed by our predictive analysis that LCD targets are enriched in pathways, such as those involving oxidoreductase activities, suggesting that AMPK may also be influenced by LCD to exert certain impacts. These findings warrant further exploration in our future research endeavors.

Several limitations of this study should be acknowledged. Firstly, this study primarily concentrated on ferroptosis and oxidative stress in neuronal cells after ICH, mediated by LCD, and future research is required to investigate other mechanisms underlying LCD’s neuroprotective impacts on SBI following ICH, including its pathway across the BBB. Secondly, though our study involved a limited sample size, the results align with prior research (Fan et al., 2024) that employed similar methodologies with just three replicates. This consistency lends credibility to our findings within the existing scientific literature. To analyze group differences, we conducted a one-way ANOVA supplemented by Tukey’s post-hoc test for detailed pairwise comparisons. Although statistically significant differences were observed among the groups, the limited sample size (n = 3) raises concerns about the study’s statistical robustness and broader applicability. Such a small cohort may diminish the ability to identify genuine outcomes while heightening the potential for false-positive results. We emphasize the importance of future studies with larger sample sizes to validate and expand upon our findings.

In this study, interventional treatment for ICH pretreatment was conducted using LCD. In future research, it is intended to explore more safe treatment approaches and optimal drug concentrations for LCD by adjusting administration timing or modifying introduction conditions into the rat model, such as via oral gavage. The full pharmacokinetic profile of LCD remains to be characterized. Bioisosteric replacements and scaffold transformations represent critical strategies in drug optimization, as they may enhance LCD’s aqueous solubility, metabolic stability, and pharmacological effect—though further research is needed to confirm their pharmacological effects.

**FIGURE 7:**
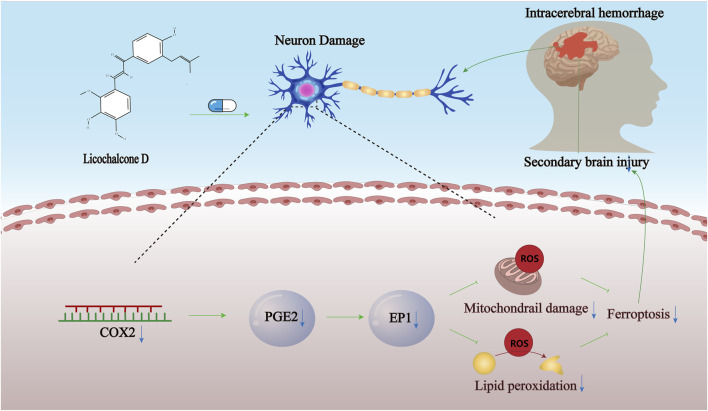
Proposed model. Propose a model demonstrating LCD’s pharmacological effects in ameliorating ferroptosis in neuronal cells following spontaneous ICH, achieved through targeted inhibition of the COX2-driven PGE2/EP1 pathway.

To summarize, it is revealed by our research that SBI following ICH can be significantly attenuated by intravenous pretreatment with LCD through the inhibition of COX2. The inhibition then tamps down the PGE2/EP1 signaling process, suppresses the generation of lipid peroxides, curbing ferroptosis in neuronal cells. Meanwhile, it lessens inflammatory responses and oxidative stress-induced damage (Figure 7).

## Data Availability

The raw data supporting the conclusions of this article will be made available by the authors, without undue reservation.
